# Effect of increased body mass index on risk of diagnosis or death from cancer

**DOI:** 10.1038/s41416-019-0386-9

**Published:** 2019-02-08

**Authors:** Puya Gharahkhani, Jue-Sheng Ong, Jiyuan An, Matthew H. Law, David C. Whiteman, Rachel E. Neale, Stuart MacGregor

**Affiliations:** QIMR Berghofer Medical Research Institute, Brisbane, QLD Australia

**Keywords:** Cancer epidemiology, Cancer genetics

## Abstract

**Background:**

Whether body mass index (BMI) is causally associated with the risk of being diagnosed with or dying from any cancer remains unclear. Weight reduction has clinical importance for cancer control only if weight gain causes cancer development or death. We aimed to answer the question 'does genetically predicted BMI influence my risk of being diagnosed with or dying from any cancer'.

**Methods:**

We used a Mendelian randomisation (MR) approach to estimate causal effect of BMI in 46,155 white-British participants aged between 40 and 69 years at recruitment (median age at follow-up 61 years) from the UK Biobank, who developed any type of cancer, among whom 6998 died from cancer. To derive MR instruments for BMI, we selected up to 390,628 cancer-free participants.

**Results:**

For each standard deviation (4.78 units) increase in genetically predicted BMI, we estimated a causal odds ratio (COR) of 1.07 (1.02–1.12) and 1.28 (1.16–1.41) for overall cancer risk and mortality, respectively. The corresponding estimates were similar for males and females, and smokers and non-smokers.

**Conclusions:**

Higher genetically predicted BMI increases the risk of being diagnosed with or dying from any cancer. These data suggest that increased overall weight may causally increase overall cancer incidence and mortality among Europeans.

## Background

High body mass index (BMI) is strongly associated with increased risk of various cancers.^1,2^ However, observational studies have reported inconsistent findings for some cancer types, with some suggesting that high BMI may even protect against breast^1^ and prostate cancer.^3^ It would be of broad interest to know how BMI would affect overall cancer risk. However, causality cannot be reliably inferred in observational studies due to issues such as confounding and reverse causation. Randomised controlled trials (RCT) would circumvent these biases but are frequently impractical to conduct. Mendelian randomisation (MR) uses genetic variants as instrumental variables to investigate whether an exposure is causally associated with an outcome of interest.^4,5^ As alleles are assigned randomly at meiosis, MR is analogous to a 'natural RCT'.^6^

Previous MR studies suggested that BMI has a different magnitude and direction of association with different cancers.^7–10^ For instance, higher BMI was reported to be causally associated with colorectal and oesophageal cancers,^7,8^ protective for breast cancer,^10^ and to have no causal effect on prostate and high-grade serous ovarian cancers.^11,12^

Given the inconsistency in the association of BMI with risk of different cancers (risk increasing, null, or even protective effects), and considering that people may not be aware of which cancer type they may develop, one interesting perspective to evaluate is whether BMI is causally associated with an overall increase (net effect) in cancer risk and/or mortality. Our similar study on height^13^ highlighted how the overall cancer risk can be a useful phenotype to understand a 'net effect' on cancer burden regardless of cancer type.

A previous MR study did not find evidence for an association between BMI and overall non-skin cancer risk in a Danish population.^14^ However, that study was statistically underpowered to make a clear statement on causality as the resultant estimates lacked precision. With a much larger sample size, we sought to identify whether BMI is causally associated with overall cancer risk, as well as cancer mortality using an MR approach. We leverage the UK Biobank cohort, expanding the set of genetic instruments available for MR and apply these to a large set of cancer diagnoses and deaths. Our results help answer the question 'does being overweight have a “net effect” on my risk of being diagnosed with or dying from cancer'; answering this issue has huge public health implications and can help inform public attitudes to obesity.

## Methods

### UK Biobank data

The UK Biobank (UKBB) is a large population-based cohort consisting of over 500 K participants aged between 37 and 73 years, recruited from 2006 to 2010 in the United Kingdom, with information collected for a series of traits ranging from anthropometric measurements and dietary behaviour to medical conditions. Data collection and genotyping details for UKBB have been described by Bycroft and colleagues.^15^ Approximately 488,000 participants were genotyped for a combined total of 805,426 markers using custom-designed Affymetrix UK BiLEVE Axiom or UK Biobank Axiom arrays (Affymetrix Santa Clara, USA). Following standard quality control (QC) and genotype phasing, approximately 96 M variants were imputed to Haplotype Reference Consortium (HRC) and UK10K haplotype resources as reference panels.^16,17^​ Imputed single nucleotide polymorphisms (SNPs; genetic markers with single nucleotide variations) with minor allele frequency (MAF) > 0.001 and imputation INFO score (accuracy score) > 0.6 were retained for the analysis. We only used ~40 M​ ​HRC-​imputed SNPs​ for this study due to the QC issues with the non-HRC imputed variants reported by UKBB.

Among the genotyped individuals that passed QC, ​409,694 participants were identified as individuals of white-British ancestry through self-report and genetics. Among those who did not report themselves as white-British, a substantial number had reported their ancestry as 'Irish' or 'any other white background' while ​their first two genetic principal components put them among those classified as white-British above (Supplementary Figure 1). To maximise our total sample size, we also included these individuals in our analyses, making our overall (final) genotyped white-British sample size 438,870 participants​.

### Cancer diagnosis and cancer death

**Fig. 1:**
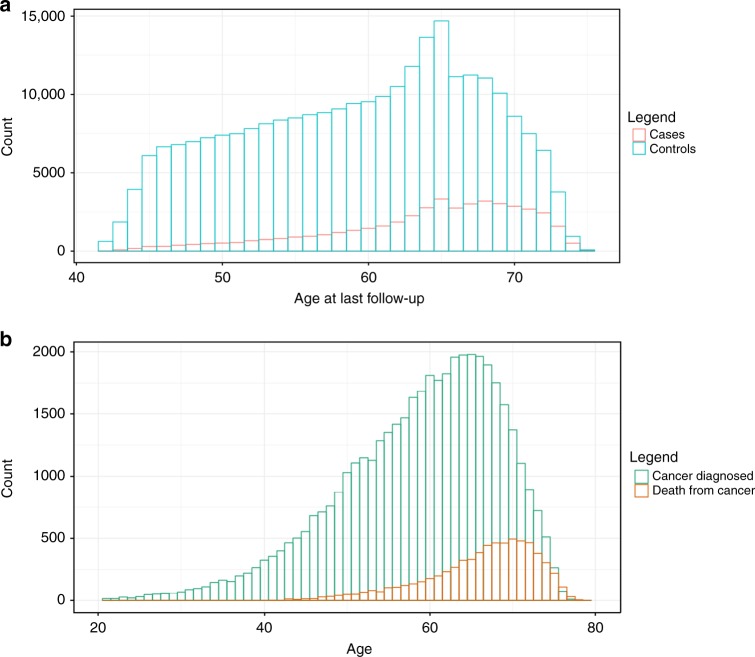
Age distribution in the UK Biobank (UKBB) participants that were included in this study. **a** Distribution of age at last follow-up in the overall cancer Mendelian randomisation (MR) study. **b** Distribution of age of first diagnosed cancer and age of death from cancer

Cancer outcomes were ascertained based on cancer registry and hospital records as previously described.^13^ Supplementary Tables 1 and 2 summarise the definition of cancer cases and controls. Distribution of age and sex within the cancer cases are shown in Table 1 and Fig. 1. PLINK (v2.0 alpha)^18^ was used to exclude related individuals with (πˆ) of > 0.2 within, as well as between cancer and control subsets.^13^ Briefly, 46,155 cases with cancer (any type, excluding keratinocyte cancers) defined based on the ICD-10 (International Statistical Classification of Diseases and Related Health Problems 10th Revision) classification, and 264,638 healthy controls without cancer, benign or in situ tumours were selected for this study. Of 46,155 cancer cases, 23,352 were prevalent cases (defined as those diagnosed prior to the UKBB recruitment date), 20,865 were incident cancer cases (defined as those diagnosed after the UKBB recruitment date; across our total sample follow-up was for between 2 and 8 years, median 4) and 1938 individuals did not have accurate diagnosis date available.

**Table 1:** Descriptive characteristics of cancer cases in UKBB that were included in this study

Cancer type	ICD-10	Cancer diagnosis			Cancer mortality		
		Females	Males	Total	Females	Males	Total
Stomach and oesophageal	C15/16	288	749	1037	136	414	550
Colorectal	C18/20/21	1974	2571	4545	327	507	834
Pancreatic	C25	248	294	542	199	248	447
Lung	C34	971	1087	2058	597	731	1328
Melanoma	C43	1748	1323	3071	71	111	182
Breast	C50	11,703	-	11,703	768	-	768
Endometrial	C53/54	1938	-	1938	143	-	143
Ovarian	C56	1031	-	1031	270	-	270
Prostate	C61	-	7532	7532	-	543	543
Kidney	C64	379	689	1068	65	163	228
Lymphoid	C81-96	1556	2102	3658	248	431	679
Overall cancer		24,989	21,166	46,155	3164	3834	6998

Sample size listed here are already trimmed for genetic relatedness. The sum of the number of participants for each cancer type reported in this table does not add up to the number of the overall cancer cases since this table only shows the cancer types that were included in the cancer-specific analyses. In addition, the overall cancer set captures a smaller set of participants than the sum of the individual cancers due to two reasons: (1) people with multiple cancer incidence are only presented once (for the earliest cancer diagnosed) in the overall cancer set, and (2) combining all cancer sets requires the related people between the individual cancer sets to be removed

*UKBB* UK Biobank, *ICD-10* International Statistical Classification of Diseases and Related Health Problems 10th Revision

For cancer death, according to the UK Death Registry, of the 14,417 death records among the UKBB participants, 7348 have died from cancer (ICD-10 cancer definitions). We used information from the UKBB field IDs 40001 (Underlying (primary) cause of death: ICD-10—Death register) and 40002 (Underlying (secondary) cause of death: ICD-10—Death register) to ensure that cancer was the main cause of death in these individuals. We also removed individuals who had inconsistent reports on cancer status between the UK cancer registry and death registry. Hence, we selected 6998 people who died from cancer and 270,342 healthy controls for the mortality analysis.

We selected 390,628 cancer-free white-British participants with BMI data (constructed from Seca 202 measured standing height (UKBB Data-Field ID: 50) and weight (UKBB Data-Field ID: 21002) measured during the initial Assessment Centre visit). Individuals with cancer were excluded to avoid reverse causality or overfitting bias in our MR estimates, which can happen as a result of sample overlap between the BMI and cancer case dataset.

### Association testing

We assessed associations between SNPs and overall/specific cancer status adjusted for age, sex and the first 10 principal components, through logistic regression under an additive genetic model, implemented in PLINK version 2.0 alpha.^19^ Similarly, the association of SNPs with cancer mortality (comparison of healthy individuals against patients who died from cancer) were evaluated through logistic regression using a similar procedure.

Quantitative association analysis for BMI was performed using a linear mixed model framework to account for cryptic relatedness and population stratification in the UKBB samples using BOLT-LMM version 2.3^20^ adjusted for age and sex. We used a sparse set of 360,087 genotyped SNPs across the autosomes to estimate the Bayesian Gaussian mixture prior to characterising the random-effects genetic component, which is used by BOLT-LMM to adjust for cryptic relatedness and population stratification. The infinitesimal model in BOLT-LMM was used to generate genome-wide association statistics.

### Instrumental variable analyses

We used the variants that were associated with BMI at a *P*-value < 1 × 10^-8^ in UKBB as instrumental variables. To ensure that the variants were independent, we pruned the variants in linkage disequilibrium (LD; associated alleles at different genomic locations) *r*^2^ = 0.01 using a 10 Mb window. The number of SNPs and phenotypic variance explained by the SNPs are shown in Table 2.

**Table 2:** Summary phenotypic variance explained by SNPs used in MR analyses for BMI

Trait	Discovery sample size	Relevant UKBB fields	Number of SNPs	*r* ^2a^	Description
BMI	390,628	UKB Field ID 50	520	0.07	SNP association adjusted for age and sex.
Filtered BMI	390,628	UKB Field ID 50	377	0.04	A subset of the BMI SNPs that are not associated (with *P* > 1e–5) to potential confounders (i.e., smoking phenotypes, alcohol intake, coffee/tea, height). For sensitivity analyses.

SNPs associated with the trait at *P*   < 1e–8 were used as instruments. SNPs were pruned for LD at *r*^2^  < 0.01 to ensure strict independence between instruments

*UKBB* UK Biobank, *BMI* body mass index, *MR* Mendelian randomization

^a^*r*^2^; proportion of phenotypic variance explained by SNPs. The phenotypic variance of trait Y explained by SNPs were calculated based on $\mathop {\sum }\limits_i 2p_i\left( {1 - p_i} \right)\beta _i^2/Var(Y)$, where _*pi*_ and *β*_i_ refers to the minor allele frequency and the magnitude of association of the *i*-th SNP instrument on trait *Y*

We used the Wald-type ratio estimator^21^ to derive a causal estimate based on the ratio between the estimated magnitude of association of BMI-associated instruments with measured BMI and the estimated magnitude of association of that SNP with cancer outcomes. These estimates were then combined using a fixed-effects inverse variance-weighted model.

### Sensitivity analyses

For a MR analysis to be valid, several assumptions must be satisfied. First, the genetic variants used as the instrumental variable must be strongly associated with the risk factor of interest (*F*-statistics > 10). This assumption is satisfied by design as we only adopted genome-wide significant SNPs as instruments. The second assumption requires that the genetic variants are not associated with any confounders of the association between the risk factor and the outcome (SNP-pleiotropy). To assess this assumption, we repeated the analyses by removing a subset of SNPs that were associated with potential confounders. These were identified by having a Bonferroni-corrected *P* < 1×10^-5^ for 520 SNPs tested in nine traits; smoking (four traits), alcohol intake (two traits), coffee/tea consumption (two traits), and height (one trait). In addition, to test for the presence of directional pleiotropy, we used two approaches: (1) we created funnel plots for the MR estimates for all SNPs used as instrumental variable, where asymmetry in the funnel plots indicates directional pleiotropy; and (2) we used the MR Egger regression approach where a non-zero intercept indicates presence of directional pleiotropy.^22^ The third assumption requires the genetic variants to be associated with the outcome (cancer risk) only through the risk factor of interest (BMI). Although the third assumption is difficult to assess directly, we employed a series of sensitivity analyses described in the Supplementary Methods, to ensure that our MR results were not biased due to including invalid instruments in the analysis.

To investigate whether the effect of BMI on cancer risk and mortality differs according to smoking status, we also performed the MR analyses by stratifying cancer cases to smokers and non-smokers. In addition, to ensure that our overall cancer findings were not largely driven by prevalent or incident cancer cases, we also estimated the causal effects in each group separately.

## Results

**Fig. 2:**
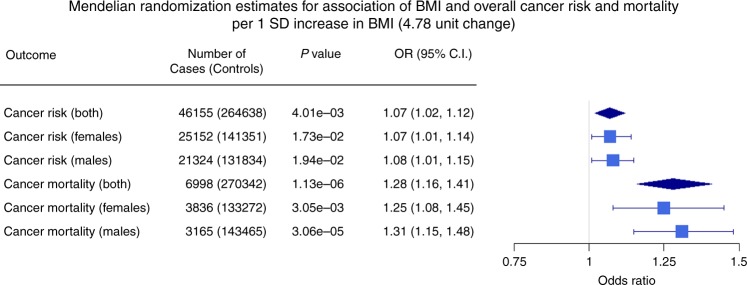
Mendelian randomisation estimates for association of body mass index (BMI) and overall cancer risk and mortality. The estimates are given per 1 SD increase in BMI (4.78-unit change). OR odds ratio

In our primary analysis, we used 46,155 cancer cases from UK Biobank, together with 264,638 healthy controls. For cancer mortality, we considered 6998 individuals who died from cancer. The estimated causal odds ratio (COR) for overall cancer risk per standard deviation (SD) (4.78-unit change) increase in genetically predicted BMI was 1.07 (95% confidence interval (CI): 1.02–1.12). Similar estimates were obtained for female and male separately (Fig. 2). The CORs for overall cancer mortality was estimated to be 1.28 (95% CI: 1.16–1.41), with little difference between females and males (Fig. 2). The results were essentially unchanged after removing SNPs associated with any potential confounders (the COR was 1.06, 95% CI: 1.01–1.13 for cancer risk and 1.27, 95% CI: 1.12–1.43 for cancer mortality, additional robustness tests revealed no evidence for pleiotropy, Supplementary Methods, Supplementary Tables 3 and 4).

The CORs for overall cancer risk in smokers and non-smokers were 1.07 (95% CI: 1.02–1.13) and 1.04 (95% CI: 0.97– 1.10), respectively. These data indicate that the causal estimate of BMI on cancer risk is not significantly different between smokers and non-smokers (*P* = 0.43).

We also repeated our MR analyses for prevalent (*n* = 23,352) and incident (*n* = 20,865) cancers separately. The CORs for overall cancer risk per SD increase in BMI were 1.04 (95% CI: 0.97–1.10) and 1.11 (95% CI: 1.05–1.18) for prevalent and incident cancers, respectively. Although the COR was larger for incident cancers, it was not significantly different from that of the prevalent cancers (*P* = 0.11) with the 95% CIs overlapping between the two groups.

**Fig. 3:**
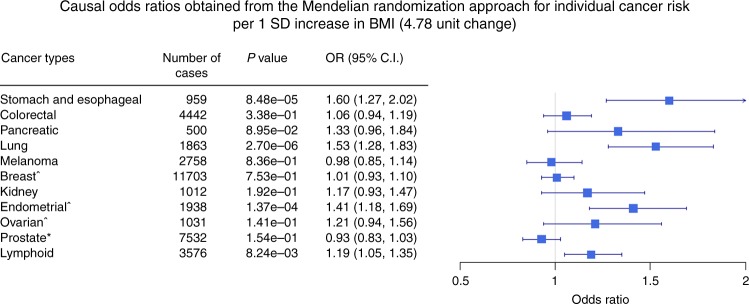
Causal odds ratios obtained from the Mendelian randomisation approach for individual cancer risk. The estimates are given per 1 SD increase in body mass index (BMI; 4.78-unit change). OR odds ratio

To ensure that the overall cancer results were not driven by a specific type of cancer, we also performed MR analysis for the risk of each cancer separately. As illustrated in Fig. 3, genetically predicted increased BMI showed significant evidence of causality for cancers of the stomach, oesophagus, lung, endometrium, and lymphoid system. Although increased BMI did not show a significant causal effect for the other cancers tested, the majority had a positive causal estimate with a wide 95% CI overlapping the null effect (Fig. 3), suggesting that repeating this analysis in a larger sample size for those cancers may reveal a significant positive causal relationship. By contrast, melanoma and prostate cancer had negative causal estimates although the results were not significantly different from the null. We did not perform cancer-specific analyses for cancer mortality due to sample size limitations (Table 1).

We also investigated whether waist–hip ratio is causally associated with overall cancer risk and mortality independent of BMI (Supplementary Methods). We did not find any evidence of significant association although we cannot rule out a small effect (Supplementary Results).

## Discussion

We found evidence of a causal relationship between BMI and overall cancer risk and mortality using a MR approach. Our results suggest that overall an increase in BMI causally increases risk of developing and dying from cancer, if we consider cancer as one clinical outcome. There was no evidence for a difference in effect between males and females. We found no evidence that the effects differed by smoking status, contrary to early reports.^23^

Our cancer-specific analyses showed that increased BMI is causally associated with increased risk of some types of cancer (e.g., cancers of stomach, oesophagus, and endometrium), but we did not observe a statistically significant association with other cancers. However, our statistical power was quite low for a conclusive interpretation of the non-significant individual cancer results, and we cannot exclude the possibility of high BMI conferring a small-to-modest increased risk of cancers such as colorectal and pancreatic or a (marginal) protective effect on cancers such as prostate cancer. Furthermore, due to sample size we did not attempt to split cancers by subtype.

Our null MR result for BMI on breast cancer (COR 1.01., 95% CI: 0.93–1.10) contradicts a previous MR study showing a protective effect.^10^ The first possible reason for discrepancy is that we used a much larger set of SNPs, explaining more variation in BMI. Second, our breast cancer phenotype definition differed; the previous breast study considered the full age range, but our study only includes younger individuals (Fig. 1).

A previous MR study concluded that BMI was not causally associated with cancer risk, although the point estimate was similar in magnitude to that reported here (OR = 1.08, 95% CI: 0.8–1.45 for a five-unit increase in genetically determined BMI); for that study, the CI was too wide to be conclusive.^14^ That study used only five SNPs as the instrumental variable, explaining only 0.4% of the phenotypic variance in BMI whereas ours explained 7% of the phenotypic variance using 520 SNPs. In addition, we included many more cancer cases than the previous study (46,155 vs. approximately 8000), again highlighting our increased power to obtain more precise and conclusive results. Moreover, we also investigated the effect of BMI on cancer mortality in this study.

In our analysis of overall cancer risk, approximately half the information came from prevalent rather than incident cases. We did not observe a significant difference for the effect of BMI on prevalent and incident cancers suggesting that it is unlikely that our overall finding is largely driven by sampling of different cancers. However, we may have under-sampled prevalent cases for cancers with very poor survival such as oesophageal, lung, and pancreatic cancer, because the prevalent cases captured would be predominantly those with a diagnosis shortly prior to recruitment into UKBB. If obesity has an atypically large effect among these under-sampled cancers, the true effect of obesity on overall cancer risk may be underestimated—further reinforcing the potential adverse effect of BMI on cancer susceptibility. In Fig. 2, some of the particular cancers with poor survival do show a large effect of obesity on risk, although the CIs for particular cancers are relatively wide as these cancers constitute only a small minority of the total number of cancer cases.

Our study is a major improvement of the previous investigation given the large sample sizes we used to calibrate BMI instruments, as well as aggregating more cancer cases. This allows us to assess moderate causal effect with reasonable statistical power (instrument strength *r*^2^ ~7%). In addition, we also demonstrated a potential adverse connection between BMI and cancer mortality via MR. We used a homogenous population with all the participants being of white-British ancestry with consistent phenotyping for cancer based on the ICD-10 definition, as well as for BMI measurements. Finally, we included most cancer cases (except keratinocyte cancers) captured in the UKBB population, which, along with the other strengths of this study, is very likely translatable to the general population given the distribution of cancer types in this study.

Our study has several limitations. First, we did not investigate the causal effect of obesity as a dichotomous risk factor (obese vs non-obese) because dichotomising a quantitative trait (here BMI) using an arbitrary threshold would likely reduce our statistical power to identify genome-wide significant loci to use as instrumental variables. Second, our MR model assumed a linear relationship between genetically predicted BMI with cancer risk and mortality as each BMI-increasing allele has a small additive effect on BMI, as well as cancer risk/mortality. The linearity assumption is controversial given the postulated 'Obesity Paradox', with the problem compounded by the fact that the evidence is typically drawn from observational studies where confounding and reverse causality can be major issues (e.g., one suggests a nonlinear dose-response relationship between BMI and postmenopausal breast cancer^24^ but interpreting such an observational study is difficult). A thorough investigation of the 'U-shaped' relationship between BMI and cancer outcomes would require an unascertained population sample, which is unfortunately not the case with the UK Biobank.^25^ This is due to the known 'healthy volunteer' bias in the UK Biobank recruitment process, effectively truncating/removing the extreme ends of the true BMI distribution. Third, we did not investigate the effect of BMI on cancer mortality in prevalent and incident cancers, separately. This was because we had limited sample size for cancer death arising from prevalent cancers to reliably estimate the effect of BMI. Given a smaller proportion of death came from prevalent cancers in our study (~30% of the total cancer deaths), it is unlikely that our mortality results were significantly influenced by the prevalent cancers.

Finally, an overall cancer phenotype may be rather heterogeneous considering the biological heterogeneity between cancers.^26,27^ In light of these heterogeneity, it is possible that the true effect of BMI on cancer might had been cancelled out. However, our previously published study on height,^13^ a trait less likely to be affected by reverse causality and confounding in observational studies, suggests that it is possible to detect an overall net effect even in the presence of cancer heterogeneity. We convincingly showed that our overall cancer MR estimates for height yielded very concordant results with previously published large observational studies, which adopted similar 'overall cancer' approaches to make statements on general public health.

## Conclusion

We demonstrated a genetic evidence for a causal relationship between BMI and overall cancer risk, with a larger effect observed on cancer mortality. Due to the high prevalence of obesity in the population, a small change in BMI can have huge implication on reducing burden of cancer. Although the estimated effect sizes for BMI are not large (particularly for risk), these findings provide evidence that cancer prevention strategies targeting weight control ought to be continued, given the high prevalence of obesity.

## Supplementary information

Supplementary_Materials

## Data Availability

The UK Biobank data are available through the UK Biobank Access Management System (http://www.ukbiobank.ac.uk/).
