# Supplementary material for: Effect of increased body mass index on risk of diagnosis or death from cancer
Source: Br J Cancer. 2019 Feb 8;120(5):565–70. doi: 10.1038/s41416-019-0386-9 (PMC6462026; doi:10.1038/s41416-019-0386-9)
Supplement: Supplementary file 1 — Supplementary_Materials [file 41416_2019_386_MOESM1_ESM.docx]

**Online Supplementary Materials**

**Effect of increased body mass index on risk of early diagnosis or early death from cancer**

Puya Gharahkhani, PhD*^1^; Jue-Sheng Ong, MMath*^1^; Jiyuan An, PhD^1^; Matthew H. Law, PhD^1^; David C. Whiteman, PhD^1^; Rachel E. Neale, PhD^1^, Stuart MacGregor, PhD^1^

^1^ QIMR Berghofer Medical Research Institute, Brisbane, Australia.

* These authors contributed equally to this work.

**Supplementary Methods**

**Sensitivity analyses:**

We used the R package MendelianRandomization v0.2.2[(1)](https://paperpile.com/c/JG7IAn/QF00h) to perform a series of sensitivity analyses using other MR approaches that account for violation of certain MR assumptions. These sensitivity analyses provide potentially more robust estimates but at the expense of wider confidence intervals. MR Egger regression[(2)](https://paperpile.com/c/JG7IAn/cXage) adjusts for directional pleiotropy and allows for direct association of genetic variants with outcome provided that those associations are independent of associations of genetic variants to risk factor of interest (Instrument Strength Independent of Direct Effect assumption). A non-zero intercept from MR Egger regression indicates presence of directional pleiotropy. We used the MR Egger Bootstrap approach to obtain more accurate standard errors on the MR Egger estimates. We applied the simple median approach[(3)](https://paperpile.com/c/JG7IAn/XeNny) which allows and adjusts for presence of up to 50% invalid instrumental variables, and hence, provides unbiased causal estimates even if half of the instrumental variables violate the second or the third MR assumptions. We also used related approaches including the inverse-variance weighted median approach as well as the penalized weighted median approach which penalizes weights for variants with heterogeneous effects. Finally, we used the maximum likelihood approach[(4)](https://paperpile.com/c/JG7IAn/q5uQP) which accounts for uncertainty in the SNP to risk factor associations, and gives unbiased estimates even if there is sample overlap between datasets used to test SNP to risk factor and SNP to outcome associations.

**Waist-hip ratio analyses:**

Waist-hip ratio (WHR) data was constructed as a ratio of waist circumference (UKBB Data-Field ID: 48) to hip circumference (UKBB Data-Field ID: 49). Using similar inclusion criteria for BMI (see the main text), we included 390,543 participants (211,439 females and 179,104 males) with WHR data in this study. WHR is known to have a different genetic architecture in males and in females[(5)](https://paperpile.com/c/JG7IAn/Vk0v6); hence we performed the genetic association analyses separately for males and females to identify sex-specific instruments, adjusted for age and BMI as covariates. Hereafter, WHR_adj_ refers to WHR adjusted for BMI. The MR analyses for WHR were performed as described in the main text.

**Supplementary Results**

**Sensitivity analyses:**

The results from the sensitivity analyses using alternative MR approaches are summarized in Supplementary Tables 3 and 4. We found no evidence of directional pleiotropy (Supplementary Figure 2). The intercept from the MR egger regression was not significantly different from zero (cancer risk: 0.0014, se=0.0013, cancer mortality: -0.0005, se=0.0026). Under the assumption that instrument strength does not correlate with the SNP to outcome association (InSIDE), the MR egger approach also provides an estimate of the COR but the resultant confidence intervals are much wider than for other approaches[(2)](https://paperpile.com/c/JG7IAn/cXage) (resulting in confidence intervals that overlap both 1 and our IVW estimate, for risk and for mortality, Supplementary Table 3 and 4). We also employed a range of median-based MR approaches; these offer improved robustness, at the expense of wider confidence intervals. Using 3 alternative median-based estimators, our causal estimates were similar to the IVW estimates (Supplementary Table 3 and 4). Finally, we used a maximum likelihood approach[(4)](https://paperpile.com/c/JG7IAn/q5uQP) which accounts for uncertainty in the SNP to risk factor associations; the results from this approach were very similar to those from the standard IVW approach (Supplementary Table 3 and 4).

**Waist-hip ratio analyses:**

For WHR_adj_, we stratified the analyses by sex to account for sex-specific genetic effects using SNPs that genetically predict WHR_adj_ in females and males separately. The estimated CORs for overall cancer risk per one SD increase in WHR (0.07 units change in females and 0.12 in males) were 1.02 (95% CI: 0.96 to 1.09) and 1.19 (95% CI: 0.88 to 1.59) for females and males, respectively. Similarly, the COR estimates for cancer death per SD increase in WHR were 1.04 (95% CI: 0.91 to 1.20) and 1.19 (95% CI: 0.69 to 2.03) for females and males, respectively.

Our instrument strength was high in females (303 SNPs explaining 7% of the phenotypic variance) but our sample size was halved compared to the BMI analyses, resulting in wider confidence intervals which overlapped 1. In males there were far fewer strong instruments for WHR_adj_ (75 SNPs explaining 1% of the phenotypic variance), resulting in reduced power to make a clear statement for WHR_adj_. Due to limited power for WHR_adj_, we did not stratify for smoking or by individual cancers.

**Supplementary Tables:**

**Supplementary Table 1 as previously reported**[(6)](https://paperpile.com/c/JG7IAn/7kNK): Inclusion criteria for case and control definition for overall cancer risk and mortality MR study.

| UKB Field ID | Description | Case/Control Criteria |
| --- | --- | --- |
| 134 | (Number of self-reported cancers) | if there is at least one question with a value > 0 then it is excluded from the control set |
| 2453 | (Cancer diagnosed by doctor Medical conditions) |  |
| 20001 | (Cancer code, self-reported) | if there is at least one question with a value which is not "NA" then it is excluded from the control set |
| 20007 | (Interpolated Age of participant when cancer first diagnosed Medical conditions) as above |  |
| 40001 | (Underlying (primary) cause of death: ICD10 - Death register) |  |
| 40006 | (Type of cancer: ICD10 - Cancer register) |  |
| 40007 | (Age at death - Death register) |  |
| 40008 | (Age at cancer diagnosis - Cancer register) |  |
| 40011 | (Histology of cancer tumour - Cancer register) |  |
| 40012 | (Behaviour of cancer tumour - Cancer register) |  |
| 40013 | (Type of cancer: ICD9 - Cancer register) |  |
| 84 | (Cancer year/age first occurred - Medical conditions) |  |
| 40009 | (Reported occurrences of cancer - Cancer register) |  |
| 40006 | (Type of cancer: ICD10 - Cancer register) | if its ICD10 code starts with "C" or "D" then exclude from the control set. If the ICD10 code starts with "C" but is not "C44" (i.e. not BCC/SCC cases), then it is classified as a case. |
| 41202 | (Diagnoses - main ICD10 - Summary Information (diagnoses)) |  |
| 41204 | (Diagnoses - secondary ICD10 - Summary Information (diagnoses)) |  |
|  | *Summary of procedure: Control* | Individuals did not report any instance of cancer diagnosis - whether through self-report or clinical diagnosis. |
|  | *Summary of procedure: Cases* | Individuals reported instances of cancer diagnosis validated through ICD10 codes starting with "C". However, individuals with C44 were excluded. |

Similar criteria were used for the cancer mortality study, mainly we additionally included field ID 40001 and 40002 to validate that cause of death for participants in the study were cancers.

**Supplementary Table 2 as previously reported**[(6)](https://paperpile.com/c/JG7IAn/7kNK): Criteria for cancer type classification based on ICD10 diagnosis.

| Cancer type grouping | ICD Groups | ICD10 prefix of cancer type | Comments |
| --- | --- | --- | --- |
| Stomach, esophageal | C15-C26 | C15,C16 |  |
| Colorectal | C15-C26 | C18,C20,C21 |  |
| Pancreatic | C15-C26 | C25 |  |
| Lung | C30-C39 | C34 |  |
| Melanoma | C43-C44 | C43 |  |
| Breast | C50-C50 | C50 | Females only |
| Kidney | C64-C68 | C64 |  |
| Endometrial | C51-C58 | C53,C54 | Females only |
| Ovarian | C51-C58 | C56 | Females only |
| Prostate | C60-C63 | C61 | Males only |
| Lymphoid | C81-C96 | C81-C96 |  |

Grouping of cancer cases were based on ICD10 diagnosis codes from UKB data-field (40006,41202,41204). For individuals diagnosed with multiple ICD10 cancer codes, the individual will be indexed in all of the cancer types he/she is diagnosed with.

**Supplementary Table 3.** This table summarizes the sensitivity analysis results obtained from the MR approaches that account for presence of invalid instrumental variables. The causal ORs and the 95% CIs are reported for overall cancer ***risk*** per standard deviation (4.7 unit change) increase in BMI. The same SNPs (N=520) were used as instrumental variable for all these methods as well as the inverse variance weighted method that we used to report the MR results in the main text.

| **Method** | **Causal OR** | **95% confidence interval** |
| --- | --- | --- |
| IVW dropping pleiotropic SNPs | 1.06 | 1.01-1.13 |
| Maximum likelihood | 1.07 | 1.03-1.11 |
| MR Egger (bootstrap) | 1.00 | 0.90-1.111 |
| Simple median | 1.08 | 1.02-1.15 |
| Weighted median | 1.03 | 0.96-1.09 |
| Penalised weighted median | 1.04 | 0.98-1.12 |

**Supplementary Table 4.** This table summarizes the sensitivity analysis results obtained from the MR approaches that account for presence of invalid instrumental variables. The causal ORs and the 95% CIs are reported for overall cancer ***mortality*** per standard deviation (4.7 unit change) increase in BMI. The same SNPs (N=520) were used as instrumental variable for all these methods as well as the inverse variance weighted method that we used to report the MR results in the main text.

| **Method** | **Causal OR** | **95% confidence interval** |
| --- | --- | --- |
| IVW dropping pleiotropic SNPs | 1.27 | 1.12-1.43 |
| Maximum likelihood | 1.28 | 1.16-1.40 |
| MR Egger (bootstrap) | 1.26 | 0.98-1.61 |
| Simple median | 1.20 | 1.04-1.39 |
| Weighted median | 1.23 | 1.06-1.43 |
| Penalised weighted median | 1.19 | 1.03-1.37 |

**Supplementary Figures**

**Supplementary Figure 1**: Plot of ancestral principal component values (PC1-PC2) in UK Biobank participants (N=487,910). The red eclipse represent the bound of PC1-PC2 values based on self-reported white-British participants. Samples with PC1-2 values within the eclipse are hence categorized as individuals of white-British ancestry for our analyses.


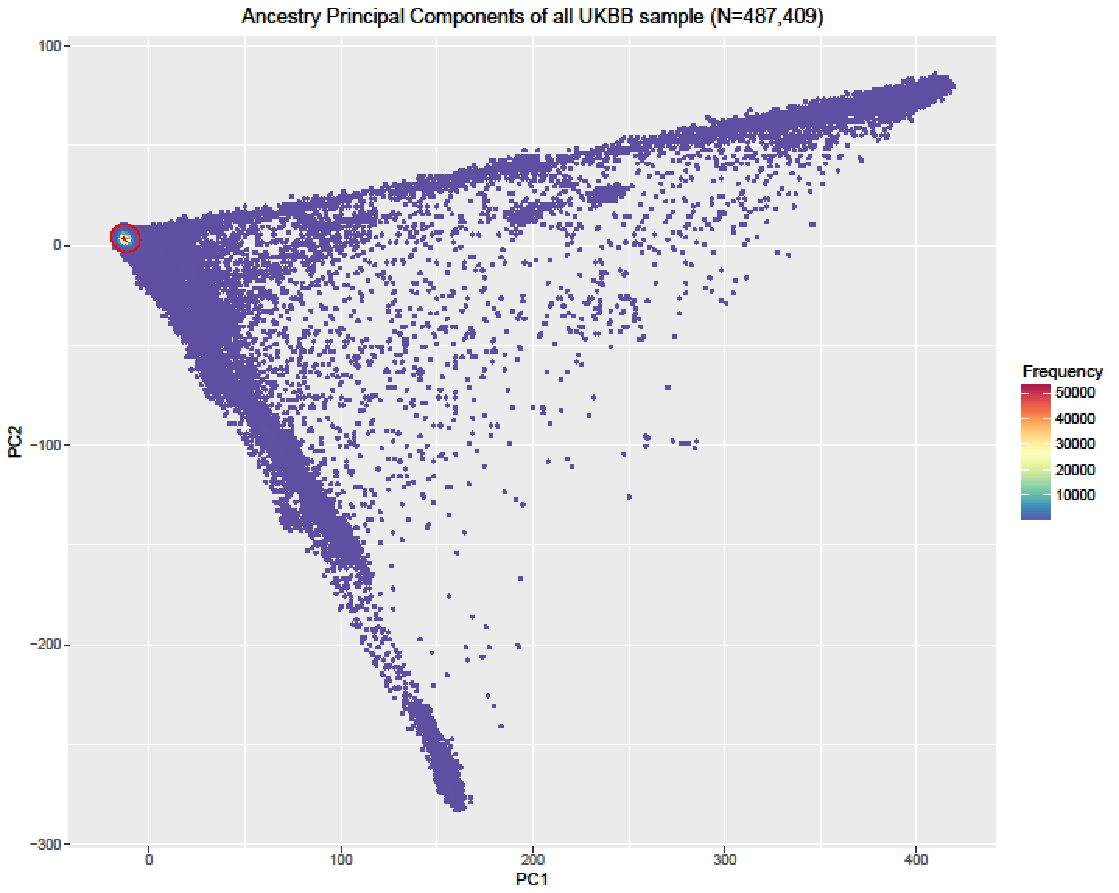


**Supplementary Figure 2.** Funnel plots for the cancer risk and mortality MR estimates. Instrumental variable (IV) estimates (X axis) are plotted against the inverse of standard error of the IV estimates (Y axis) for each SNP. The vertical lines indicate the causal estimates obtained through MR Egger regression and inverse variance weighted approaches. Asymmetry in the funnel plots indicates directional pleiotropy. a) Funnel plot for all cancer risk. b) Funnel plot for all cancer mortality.

a)

**
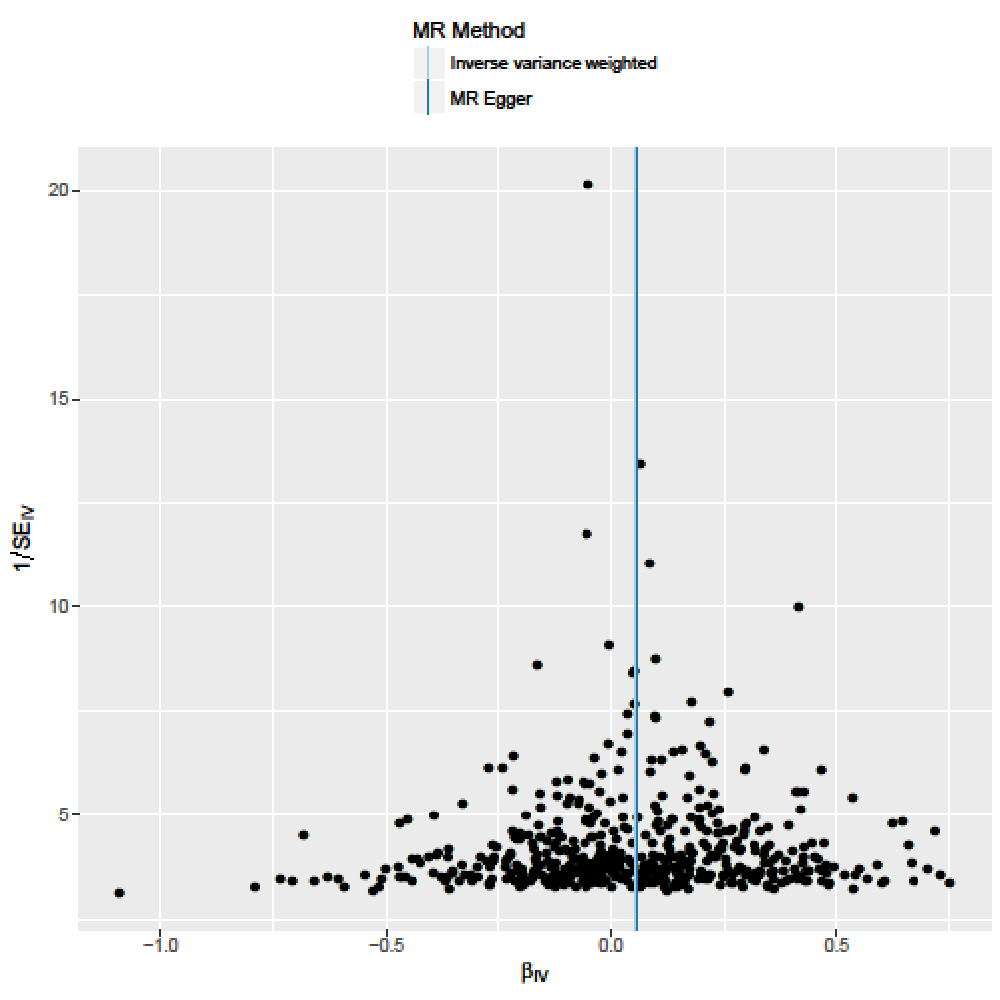
**

b)

**
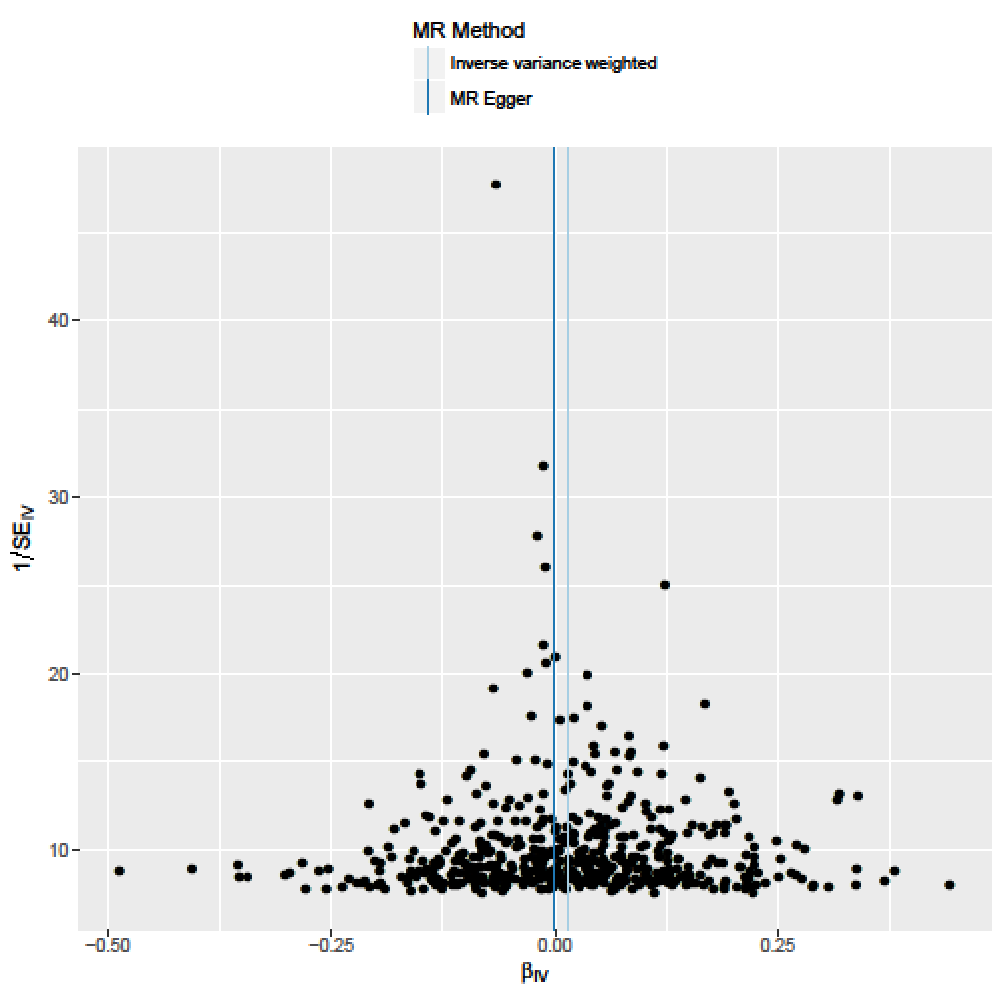
**

**References**

^1.^ [Yavorska OO, Burgess S. MendelianRandomization: an R package for performing Mendelian randomization analyses using summarized data. Int J Epidemiol [Internet]. 2017 Apr 7; Available from:](http://paperpile.com/b/JG7IAn/QF00h) <http://dx.doi.org/10.1093/ije/dyx034>

2. [Bowden J, Davey Smith G, Burgess S. Mendelian randomization with invalid instruments: effect estimation and bias detection through Egger regression. Int J Epidemiol. 2015 Apr;44(2):512–25.](http://paperpile.com/b/JG7IAn/cXage)

3. [Bowden J, Davey Smith G, Haycock PC, Burgess S. Consistent Estimation in Mendelian Randomization with Some Invalid Instruments Using a Weighted Median Estimator. Genet Epidemiol. 2016 May;40(4):304–14.](http://paperpile.com/b/JG7IAn/XeNny)

4. [Burgess S, Butterworth A, Thompson SG. Mendelian randomization analysis with multiple genetic variants using summarized data. Genet Epidemiol. 2013 Nov;37(7):658–65.](http://paperpile.com/b/JG7IAn/q5uQP)

5. [Randall JC, Winkler TW, Kutalik Z, Berndt SI, Jackson AU, Monda KL, et al. Sex-stratified genome-wide association studies including 270,000 individuals show sexual dimorphism in genetic loci for anthropometric traits. PLoS Genet. 2013 Jun;9(6):e1003500.](http://paperpile.com/b/JG7IAn/Vk0v6)

6. [Ong J-S, An J, Law MH, Whiteman DC, Neale RE, Gharahkhani P, et al. Height and overall cancer risk and mortality: evidence from a Mendelian randomisation study on 310,000 UK Biobank participants. Br J Cancer. 2018 May;118(9):1262–7.](http://paperpile.com/b/JG7IAn/7kNK)
